# Causes of Bad Taste and Odor in the Mouth

**Published:** 1898-02

**Authors:** 


### Causes of Bad Taste and Odor in the Mouth.

The notion existing among the laity, and also among physicians, that gastric disturbances are almost exclusively responsible for bad taste in the mouth, is wrong, says Dr. Herzfeld, in the *Therap. Monats.* (XI, 1). Only second to affections of the stomach as an etiological factor, are the tonsillar crypts, in which there accumulate mucus, dead epithelial cells and particles of decomposing food. These cheesy accumulations sometimes come out spontaneously and have a fecal odor. The reason they are so frequently overlooked is because they are concealedby the anterior pillars of the fauces and can be seen by only using a retractor. The treatment consists in removing the tonsils or in slitting the crypts open with a narrow, curved knife. Among the other causes of bad taste the author enumerates: carious teeth, inflammations of the mouth and the throat, adenoids, ozena, suppuration in the nose and accessory cavities, suppurative inflammation of the ear, and lastly, the cause may be of a nervous nature (paræsthesia gustatoria).

